# Long-Term Costs of Ischemic Stroke and Major Bleeding Events among Medicare Patients with Nonvalvular Atrial Fibrillation

**DOI:** 10.1155/2012/645469

**Published:** 2012-10-02

**Authors:** Catherine J. Mercaldi, Kimberly Siu, Stephen D. Sander, David R. Walker, You Wu, Qian Li, Ning Wu

**Affiliations:** ^1^Center for Epidemiology and Database Analytics, United BioSource Corporation, 7101 Wisconsin Avenue, Suite 600, Bethesda, MD 20814, USA; ^2^Health Economics and Outcomes Research, Boehringer Ingelheim Pharmaceuticals, Inc., 900 Ridgebury Road, Ridgefield, CT 06877, USA; ^3^Economic Analysis and Solutions, United BioSource Corporation, 7101 Wisconsin Avenue, Suite 600, Bethesda, MD 20814, USA

## Abstract

*Purpose*. Acute healthcare utilization of stroke and bleeding has been previously examined among patients with nonvalvular atrial fibrillation (NVAF). The long-term cost of such outcomes over several years is not well understood. *Methods*. Using 1999–2009 Medicare medical and enrollment data, we identified incident NVAF patients without history of stroke or bleeding. Patients were followed from the first occurrence of ischemic stroke, major bleeding, or intracranial hemorrhage (ICH) resulting in hospitalization. Those with events were matched with 1–5 NVAF patients without events. Total incremental costs of events were calculated as the difference between costs for patients with events and matched controls for up to 3 years. *Results*. Among the 25,465 patients who experienced events, 94.5% were successfully matched. In the first year after event, average incremental costs were $32,900 for ischemic stroke, $23,414 for major bleeding, and $47,640 for ICH. At 3 years after these events, costs remained elevated by $3,156–$5,400 per annum. *Conclusion*. While the costs of stroke and bleeding among patients with NVAF are most dramatic in the first year, utilization remained elevated at 3 years. Cost consequences extend beyond the initial year after these events and should be accounted for when assessing the cost-effectiveness of treatment regimens for stroke prevention.

## 1. Introduction

Atrial fibrillation (AF) affects more than 3 million Americans and is characterized by an irregularly irregular heart rhythm, often with a rapid heart rate that may result in blood clots, shortness of breath, and overall weakness [1]. Primarily a disease of the elderly, the prevalence of AF doubles with each decade of life after the age of 60 years and occurs in about 10% of the population by 80 years [2, 3]. AF is associated with a five-fold increase in the risk of ischemic stroke and accounts for 15%–20% of all strokes [3]. Anticoagulation therapy with warfarin is recommended for patients with AF who are at moderate to high risk of stroke based on clinical guidelines [4], but it has been well documented that proper dosing and close monitoring are imperative to attain the correct level of anticoagulation to balance the risk of stroke without introducing a disproportionate risk of bleeding [5–7].

Costs to manage AF in the USA are estimated at $6.65 billion, including $4.88 billion in hospitalization expenses and $1.53 billion in outpatient management costs (in 2005 US$) [8]. Accounting for some fraction of these costs are expenses related to occurrence of stroke and hemorrhagic events. While numerous studies have estimated direct and indirect costs of stroke and bleeding outcomes among AF patients in the first year following the event [9–11], long-term US cost estimates have generally relied on modeling techniques extrapolating available data covering a shorter period of time. Lifetime and other long-term cost modeling estimates would be expected to be more accurate if based on data covering a period of time extended beyond 1 year [12]. Furthermore, direct estimates of stroke and bleeding costs over several years could show whether costs for patients with these events remain elevated or eventually return to baseline levels, that is, the expected cost to treat AF without stroke or major bleeding.

The objective of the current study was to quantify the direct long-term costs, up to 3 years, of both stroke and bleeding events among patients with non-valvular AF (NVAF), which comprises approximately 90% of the overall AF population [13]. To separate the costs attributable to these events from the general costs of healthcare associated with NVAF, we conducted a retrospective cohort study of Medicare beneficiaries newly diagnosed with NVAF who later developed stroke or hemorrhagic events and were matched with NVAF patients who did not experience these outcomes based on demographic and NVAF disease characteristics.

## 2. Methods

### 2.1. Data Source

The study was conducted using 1999–2009 data from the Centers for Medicare & Medicaid Services (CMS) 5% sample standard analytical files (SAF)—limited data set (LDS). In contrast to commercial claims data sources, Medicare has a large number of patients over the age of 65 years, when incidence and prevalence of AF begin to increase dramatically. The analysis used fee-for-service (FFS), nondisabled Medicare patients who were not eligible for Medicaid. The files contain final action claims with all adjustments resolved for a 5% sample of all Medicare beneficiaries in each calendar year, including inpatient, outpatient, emergency room (ER), skilled nursing facility (SNF), hospice, home health agency, durable medical equipment (DME), and carrier (formerly Part B physician/supplier) claims. As this study was a retrospective analysis of existing, deidentified claims data, an institutional review board evaluation was not applicable and therefore, not conducted.

### 2.2. Patient Identification

Patients with AF were identified by at least 1 inpatient claim or 2 outpatient claims in the same calendar quarter with International Statistical Classification of Diseases and Related Health Problems, 9th Edition (ICD-9) code 427.31. Included patients were required to have a baseline period of 1 year (4 quarters) of continuous enrollment prior to the quarter of AF diagnosis. Patients with evidence of valvular conditions during the baseline period or quarter of AF diagnosis (codes in Table 1) were excluded.

Because the stage of NVAF disease progression has an impact on medical utilization (e.g., newly diagnosed patients tend to have higher costs than prevalent patients), the study was limited to patients with incident NVAF, defined as no AF claims during the baseline period, to allow patients to be matched on duration of NVAF. Similarly, strokes and/or bleeding events prior to AF diagnosis could lead to elevated baseline costs and would bias the estimate of long-term event costs after diagnosis. Thus, patients with prevalent AF (any claim with ICD-9 diagnosis code 427.31 during the baseline period) and patients with a principal inpatient claim with a code for stroke [14, 15] or bleeding [16, 17] events (Table 1) in the baseline period (1 year prior to AF diagnosis) or quarter of AF diagnosis were excluded.

### 2.3. Matching

All incident NVAF patients without a history of ischemic stroke or major bleeding event were then followed for first ischemic stroke or major bleeding event using validated ICD-9 codes (Table 1) [14–17]. Patients with events were matched with NVAF patients without events on age group at NVAF diagnosis, gender, race, geographic region, year of NVAF diagnosis, duration of enrollment, and warfarin use. The data did not include prescription claims; therefore, warfarin therapy was inferred for patients with at least 3 prothrombin tests (ICD-9 codes V58.61, CPT codes 85610, 85611, 99363, and 99364) during a 1-year period after NVAF diagnosis. This methodology has been validated previously in the Medicare data with 89% sensitivity and 92% specificity [6].

Each patient with an event was matched to up to 5 control patients via the greedy matching algorithm [18, 19]. Allowing more than 1 control to be matched to each patient with an event has been shown to improve statistical efficiency [20, 21]. While efficiency gains are limited beyond 3 or 4 matches, the low cost of obtaining additional matches in claims data permitted us to extend the maximal number of matches to 5. The number of matches were allowed to differ for each patient with an event, since varying numbers of controls have been shown to reduce bias [18]. Any patients not matched (either unmatchable patients with events or remaining controls) were excluded from the analysis.

### 2.4. Followup

Although the primary objective was to estimate costs up to 3 years following events, we did not limit the study population to those with 3 years of followup because doing so would have biased the population toward healthier patients. As such, no minimum duration of enrollment following the initial event was required. The quarter of the stroke or bleeding event was considered the start of followup for the patient with the event and for all controls matched to that patient.

The risk of stroke and bleeding events is somewhat intertwined (e.g., prior stroke is a stated risk factor for major bleeding), and it is possible that patients with an initial stroke (or bleed) may later experience a major bleed (or stroke). To keep the costs of these events distinct, if a patient experienced a bleeding event after stroke or a stroke after a major bleed, we ended followup for this patient in the quarter prior to the alternate event. A new followup period began with the quarter of the alternate event, and utilization measures were calculated separately for patients with both events. Thus, patients (and their matched controls) were categorized by the type(s) of events they experienced during followup as (1) patients with stroke events only or whose first event was a stroke, (2) patients with bleeding events only or whose first event was a major bleed, or (3) patients with both stroke and major bleeding events. To illustrate, a patient who first experienced a stroke and then later had a major bleeding event was categorized both in the ischemic stroke group (to allow followup before the latter event to contribute to the estimation of stroke utilization) as well as in the group for patients with both events for the remainder of follow-up beginning with the subsequent bleeding event. Under this definition, some patients were double-counted across groups, although each quarter of followup was assigned to only one event group (stroke, major bleeding, or both). Patients who had an initial stroke and a major bleeding event in the same quarter (and their matched controls) contributed only to the group of patients with both events. Because patients with strokes or bleeds in the baseline period were excluded, recent history of these events had no impact on cost estimates. Recurrent strokes (a new stroke event following the initial stroke) or bleeds (a new bleeding event following the initial bleed) are an important contributor to long-term event costs and were included during followup.

Thus, followup began with the initial event of interest and ended with the first occurrence of (1) Medicare disenrollment (including patients changing from FFS to capitation, dropping Part B coverage, becoming eligible for state Medicaid services, becoming disabled, or dying), (2) for patients with stroke events, a subsequent bleeding event or for patients with bleeding events, a subsequent stroke event, or (3) 3 years after initial stroke or bleeding event. For patients with both stroke and bleeding events, the second followup will begin in the quarter of the latter event and end with either disenrollment or 3 years after the start of the second follow-up period, whichever occurs first.

### 2.5. Patient Characteristics

Basic demographic information including age, gender, race, and geographic region were assessed at the time of AF diagnosis. We also reported risk factors for both stroke and bleeding events. Risk factors were assessed during the baseline period and in the quarter of AF diagnosis. Stroke risk was quantified using the CHA_2_DS_2_-VASc score [22]. Bleeding risk was assessed using the HAS-BLED score, which had the best predictive value of several hemorrhage risk scores [23–27] in a recent assessment [28]; however, time in the therapeutic international normalized ratio (INR) range and drug use were unavailable in the data source and thus, were excluded from the HAS-BLED definition for this study. Definitions of stroke and bleeding risk factors used to calculate these scores are provided in Table 1.

### 2.6. Identification of Events

Stroke and major bleeding events were identified using principal ICD-9 codes on inpatient hospitalization claims only. Previously validated ICD-9 codes for identifying stroke [14, 15] and bleeding [16, 17] outcomes are provided in Table 1. Major bleeding events were divided into intracranial hemorrhage (ICH) and other major bleeding event, and results were produced separately for these hemorrhagic events. Because the Medicare 5% sample data only have calendar quarter reported on claims, events were recorded starting in the quarter following NVAF diagnosis.

### 2.7. Definition of Outcomes

The primary outcome was the incremental costs of stroke and bleeding events up to 3 years after the event. Costs were from a Medicare perspective (limited to Parts A and B paid amounts) and did not include oral medications or copayments from any supplemental insurance. Control patients with NVAF but no study events (stroke or major bleeding) were used to estimate the baseline cost of treating NVAF. Costs for patients with stroke or major bleeding events beyond the baseline treatment cost of NVAF were attributed to the event. Thus, the incremental costs of stroke and major bleeding were estimated as the difference in costs between patients with events and their matched controls, with cost accrual beginning in the quarter of the event and up to 3 years afterward. For example, if a patient had a stroke in the third quarter following the NVAF diagnosis, then we began summing costs for this patient and any controls matched to this patient in the third quarter after the NVAF diagnosis. Incremental costs were also broken down by costs occurring in inpatient, outpatient, ER, SNF, hospice, and home healthcare settings, as well as DME costs to determine aspects of care driving the potential cost consequences associated with management and treatment of NVAF. Other utilization measures included number of inpatient admissions and associated length of stay and number of outpatient or office visits. 

Patients with recurrent stroke, ICH, or other major bleeding events were also identified, as well as the time from the initial event until recurrence. Recurrent events were only identified in the quarter following the initial event since the dating system in the data source made it impossible to discern between separate events occurring in the same quarter.

### 2.8. Analysis

All variables will be reported descriptively as means with standard deviations and medians with ranges for continuous variables and counts with percentages for categorical measures for patients with and without study events. Cost measures were adjusted to 2011 US dollars (USD) using the medical care component of the Consumer Price Index (CPI) annual inflation measures. Total and incremental utilization variables after a stroke/bleeding event were reported for 3 months (quarter of the event), 6 months, 9 months, 12 months, 18 months, 24 months, and 36 months. Because not all patients had the full 3 years of followup, the patient denominator declined over time. Comparison of total costs for patients with events and matched controls were conducted using standard 2-sided *t*-tests assuming unequal variances and a significance level of *α* = 0.05.

Although matching should eliminate the need to adjust for all variables that were matched in the analysis of cost differentials, in order to adjust for any potential residual confounding, total incremental costs of strokes and bleeding events were adjusted for both matching variables and individual measures within the CHA_2_DS_2_-VASc and HAS-BLED scores, respectively. Multivariate adjusted costs were estimated using generalized estimating equation (GEE) models with a gamma distribution and log link function [29]. Bootstrapping techniques were employed to estimate standard errors of adjusted costs once the parameter estimate was retransformed from the log form. All analyses were conducted using SAS version 9.2 (SAS Institute, Cary, NC).

## 3. Results

A total of 445,796 patients with at least 1 inpatient or 2 outpatient claims with diagnosis codes for AF were identified in the data. After excluding patients without continuous enrollment in the baseline period (24.3%), disabled and Medicaid-eligible patients (4.6%), patients with valvular conditions (1.9%), patients with prevalent AF (9.5%), and patients with a history of stroke or hemorrhagic conditions (4.8%), 245,052 patients with NVAF was available for the study. Of these patients, 8,243 (3.4%) experienced an ischemic stroke during followup, 1,406 (0.5%) had an ICH, and 15,816 (6.5%) had other types of major bleeding events. A total of 1,291 (0.5%) patients had both an ischemic stroke and major bleeding event (either ICH or other bleeding outcome). Matching was successful for 94.5% of all patients with stroke and major bleeds, with an average of 4 matched controls for each patient with an event. Among patients with events who were matched, the average time from NVAF diagnosis to event was 30 months for all events.

Descriptive statistics of matching characteristics show that patients with events and controls were balanced on all measures (Table 2). Average age was similar for patients with ischemic stroke, ICH, and other major bleeding events at approximately 80 years. Duration of followup was at least 2 years for 44% of patients, with 29% having data for all 3 years examined. Although patients were not matched on stroke or bleeding risk, patients with events and controls had similar CHA_2_DS_2_-VASc and HAS-BLED scores (Table 3). 

Patients with both events had an average age of 81.1 years (81.3 years for matched controls), with 61.8% of patients female (62.7% controls). Warfarin use was prevalent for 37.5% of patients with both stroke and major bleeding events. CHA_2_DS_2_-VASc score averaged 4.02 points (3.80 for controls), and mean HAS-BLED was 1.37 (1.31 for controls). Approximately one-third (32.8%) of patients with both events had data for at least 2 years after the secondary event, and 19.4% had data at 3 years.

### 3.1. Ischemic Stroke Cohort

**Figure 1:**
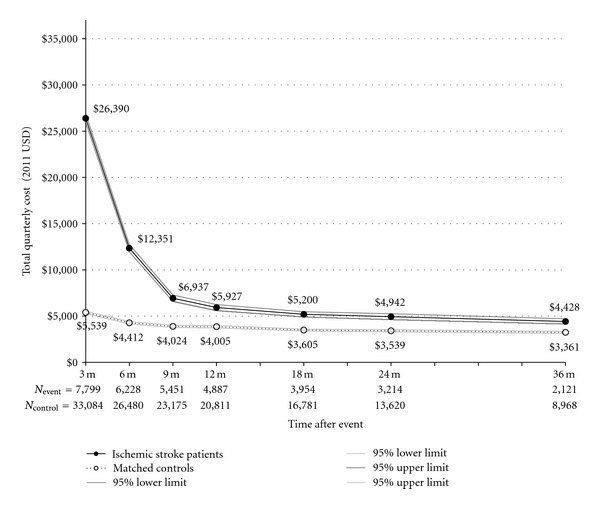
Total quarterly cost for Medicare patients with NVAF with and without ischemic stroke. Note: *P* < 0.0001 for difference in costs between patients with events and matched controls at all-time points using *t*-test assuming unequal variances and *α* = 0.05 level.

The ischemic stroke cohort consisted of 7,799 patients with stroke and 33,084 matched controls without study events. At 1 year poststroke, 62.9% of patients were still contributing data. By 2 years, 41.2% remained in the cohort, and at the end of followup 3 years after the event, 27.1% of patients were available. Among patients with stroke, 6.7% died, with 52.4% of deaths occurring in the quarter of the event. A total of 7.0% of stroke patients later went on to experience major bleeding events (either ICH or other major bleeds). Average quarterly total Medicare-reimbursed costs for patients with ischemic stroke and their matched controls without events are presented in Figure 1. The mean acute incremental cost of stroke in the quarter of the event was $20,604, and the average total 1-year cost was $32,900 more for patients with stroke than among controls. Total cumulative cost of stroke at 2 years was $36,515 and at 3 years was $38,712 for patients contributing data at these time points. In the second year after the event, total costs were $5,621 higher for stroke patients than for matched patients without events. Costs remained elevated in the third year after stroke, with an average incremental cost of $3,775. After adjusting for stroke risk factors and matching characteristics, the acute and annual incremental costs of stroke were slightly higher than in unadjusted analyses (Table 4). The largest contributors to second-year and third-year incremental costs associated with stroke were inpatient costs, SNF costs, and home healthcare costs (Table 5). In the third year after the event, patients with stroke averaged 5.2 more office visits than those without events. Recurrent ischemic stroke occurred in 6.7% of stroke patients, with an average time to recurrence of 12.0 months. The total incremental costs for patients with recurrent stroke were approximately $3,000–$4,000 higher as compared to those without recurrence in each of the first 3 years following the initial event (Table 5). 

### 3.2. Major Bleeding Cohorts

**Figure 2:**
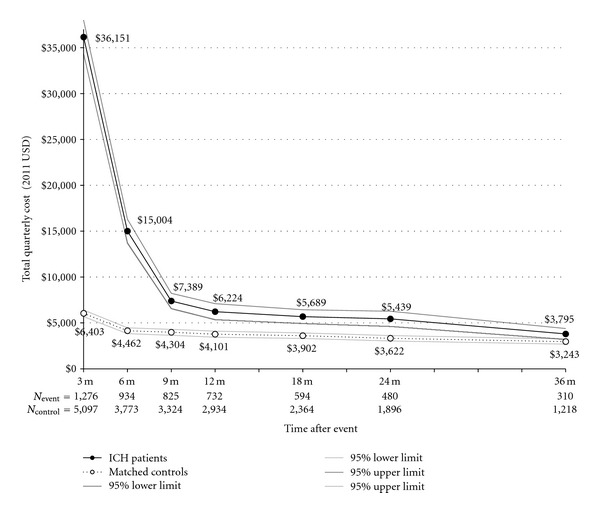
Total quarterly cost for Medicare patients with NVAF with and without ICH. Note: *P* < 0.01 for difference in costs between patients with events and matched controls at all-time points using *t*-test assuming unequal variances and *α* = 0.05 level.

A total of 1,276 patients with ICH and 5,097 matched controls were included in the study. Follow-up data was available for 57.5% of patients at 1 year after event, 37.3% at 2 years, and 24.0% after 3 years. The overall mortality rate for this cohort was 11.4%; 78.1% of deaths occurred in the quarter of the event. Among ICH patients, 4.7% subsequently had an ischemic stroke. The acute incremental cost of ICH in the quarter of the event was $29,877 (Figure 2). Total costs in the first year following ICH (including the quarter of the event) were $47,640 more for patients with events than among controls. The total cumulative cost of ICH after 2 years was $53,074 and at 3 years was $54,158. Second-year post-ICH costs were $7,910, and by the third year after the event, costs continued to be $3,156 higher than controls. Adjustment for bleeding risk factors and matching characteristics resulted in marginally higher ICH cost estimates in the first 2 years after the event and a similar value in year 3 as compared to the unadjusted results (Table 4). Inpatient, outpatient, SNF, and hospice utilization were the principal cost drivers in year 2 (Table 5). In the third year after ICH, hospice and outpatient costs were most elevated as compared to control costs. A total of 3.1% of patients with this event had a recurrent ICH. Average time to recurrence was 8.1 months. Total incremental costs for patients with recurrent ICH were approximately $7,000 more than for those without recurrence in the first year after the event, but were not elevated in the second and third years thereafter (Table 5).

**Figure 3:**
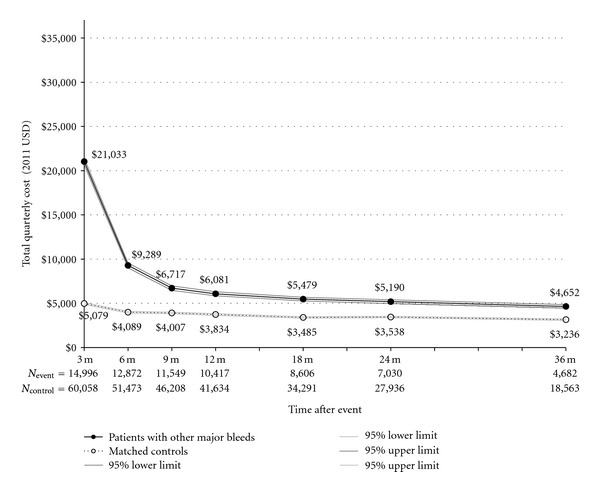
Total Quarterly Cost for Medicare Patients with NVAF with and without Other Major Bleeds. Note: *P* < 0.0001 for difference in costs between patients with events and matched controls at all time points using *t*-test assuming unequal variances and *α* = 0.05 level.

The cohort of patients with major bleeding events other than ICH had 14,996 patients with events and 60,058 controls. At 1, 2, and 3 years after the event, 69.4%, 46.6%, and 31.0% of these patients contributed data, respectively. The rate of mortality for patients with major bleeds was 4.3%, with 36.3% of those deaths taking place in the quarter of the event. Ischemic stroke followed the bleeding event in 3.1% of these patients. Patients with major bleeding events had an average acute incremental cost of $15,699 (Figure 3), with total first-year cost of $23,414 more than for matched controls. Total cumulative cost 2 years after major bleeding events averaged $28,064 and after 3 years was $31,393. The second-year and third-year incremental costs after major bleeds were $6,936 and $5,400. Similar to the ischemic stroke and ICH results, after controlling for bleeding risk factors and matching variables, estimates of the incremental costs of other major bleeds were comparable to, if slightly higher than, unadjusted figures (Table 5). Inpatient and outpatient costs were particularly elevated in the second and third years after the event (Table 4). In year 2, patients with major bleeding events had 0.5 more inpatient admissions and 11.0 additional outpatient visits as compared with control patients. Number of inpatient and outpatient visits remained increased by 0.3 admissions and 8.8 visits in the third year after the event. Recurrent major bleeding events (excluding ICH) were more common than repeat ischemic stroke or ICH events, occurring in 10.7% of stroke patients, with an average time to recurrence of 11.7 months. Patients with recurrent major bleeding events had substantially higher incremental costs in the first 3 years following the initial event as compared to those without recurrence, ranging from about $4,000–$7,000 in each year (Table 5).

### 3.3. Patients Experiencing Both Ischemic Stroke and Major Bleeding Events

Of the 1,291 patients (matched with 5,608 controls) who experienced both events, the first event was ischemic stroke for 544 (42.1%) patients, ICH for 60 (4.6%) patients, and other major bleeding for 461 (35.7%) patients. Initial stroke and major bleeding events occurred in the same quarter for the remaining 236 (18.3%) of patients. Average time from stroke to major bleeding event was 12.0 months for patients with an initial stroke. For patients with ICH as the first event, mean time to ischemic stroke was 9.8 months, and for patients with other types of major bleeds, the average time to stroke was 11.7 months. The mortality rate for patients with both types of events was 8.1%, and 43.3% of deaths occurred in the quarter of the secondary event. Total costs in the first year following the secondary event were $37,691 more for patients with events than for controls. The total cumulative cost of patients with both events after 2 years was $46.857 and at 3 years was $60,511. Second-year incremental costs were $10,519, and by the third year after the event, costs increased up to $13,512 higher than controls. After adjusting for both stroke and bleeding risk factors, as well as matching characteristics, estimates of year 1, 2, and 3 costs for patients with both ischemic stroke and major bleeding events were $42,271 (95% CI: $39,542–$45,907), $10,146 (95% CI: $6,696–$14,192), and $12,722 (95% CI: $9,287–$16,710), respectively. Recurrent events (either stroke or major bleeds) occurred in 14.2% of patients after the secondary event, with average time to recurrence of 10.5 months. Patients with recurrent events had costs $19,042 higher in the first year and just over $3,000 higher in the second and third years after the latter event as compared to those without recurrence.

## 4. Discussion

In this large population of Medicare beneficiaries with NVAF, the total direct medical costs of patients with incident ischemic stroke, ICH, and other major bleeding events were higher than for matched control patients in the first year following the event. Costs stabilized beyond the initial year after these events, but never returned to the same level as for patients with NVAF who never experienced a stroke or bleeding outcome. Even in the third year after these events, costs remained elevated by $3,000–$6,000 after adjusting for event risk factors and matching characteristics. Patients who experienced both ischemic stroke and major bleeding events suffered the highest rates of recurrence and had incremental costs of more than $10,000 in the second and third years after the secondary event as compared to controls. These results imply that the cost consequences of ischemic stroke occurring due to untreated NVAF and bleeding associated with anticoagulation treatment are observable beyond the first year after the event. This study builds on previous work estimating event costs in NVAF patients [30] by delineating between the costs attributable to new stroke and bleeding event versus expenses due to management of the underlying NVAF condition.

NVAF baseline costs were similar for all control groups. Because followup for some patients began shortly after NVAF diagnosis due to an early stroke or bleeding event, average costs for the control groups were higher in the initial quarters of the study due to the greater expense of establishing a treatment regimen for new NVAF patients versus managing patients with prevalent disease. Total costs for managing established NVAF ranged from approximately $3,000–$4,000 per quarter, confirming previous estimates of $10,100–$14,200 per year ($2,525–$3,550 quarterly) in the USA [11].

In the first year of followup, ICH was the most expensive of the events examined, followed by ischemic stroke and then other major bleeding events. Shorter-term costs for stroke and hemorrhagic events found in this study are largely consistent with prior work. We found first-year costs for ischemic stroke to be $34,772, which is within the range of estimates reported in a systematic review by Miller et al. ($24,991–$142,251) [12, 31–33]. Acute cost of major bleeding complications for patients receiving anticoagulation therapy was estimated to be $15,988 by Fanikos et al. in 2005, which is closely approximated by our total cost estimate of $16,437 in the quarter of the major bleed. To our knowledge, no previous study has reported direct cost estimates for stroke and bleeding events among NVAF patients beyond the first year after the event. By the third year after the event, patients with major bleeding events other than ICH had mean adjusted costs $5,852 higher than matched controls, as compared to $4,504 for ischemic stroke and $3,150 for ICH. This difference in long-term costs may be related to the rate of recurrence, as nearly as 11% of patients with major bleeds had a recurrent event versus 7% of ischemic stroke patients and only 3% of ICH patients. Patients with recurrent events tended to be more costly than those without recurrence, particularly those with repeat non-ICH major bleeding events. The reported differences in incremental costs among patients with and without recurrence confirms that repeat events are important cost drivers, although they do not fully explain the elevation in costs found over 3 years for patients with stroke and major bleeding as compared to those who never experienced these events, as the costs for patients with only one event were still more than $2,000–$4,000 greater than for patients without events.

Although analyses conducted using a claims database have several advantages, including large sample sizes and the ability to examine real-world treatment patterns, some limitations were present. For one, we were restricted to the data that were available in the source (i.e., missing data, limited variables, etc.). Some uncertainty exists regarding the exact timing of events because only the quarter in which the claim occurred (rather than the exact date) was available in the data. For instance, if a stroke event occurred at the end of a quarter, costs may appear to be lower than had it happened in the beginning of the quarter. Because the primary objective of this study was to examine long-term costs, the impact of such temporal issues is expected to be small. In addition, data on use of medications such as nonsteroidal anti-inflammatory drugs or antiplatelet therapies were of interest, however, this information was not available in the data source. We were able to approximate the use of anticoagulants using previously validated algorithms examining INR tests, but the laboratory value of these tests was unknown, and therefore, we had no way to assess how adequately these patients are being treated via measures such as time in the target therapeutic INR range. Similarly, INR tests administered in anticoagulation clinics or using point-of-care systems during office visits were not available, allowing for potential misclassification of warfarin exposure. It is also possible that some patients may have become disabled or eligible for Medicaid as the result of a stroke or major bleeding event. Since we were unable to fully capture costs for patients for whom Medicare was not the primary payer, followup was ended for such patients once they qualified for these services. These patients may have been sicker than patients who did not become disabled or Medicaid eligible, so cost estimates from this study may have underestimated the true cost of stroke and major bleeding events in patients with NVAF.

The study methods aimed to compare patients with and without events at a similar stage in NVAF disease progression by matching on age and time of initial diagnosis, but it is possible that some patients were initially diagnosed at a more advanced stage of disease or that some patients' disease progressed more quickly than that of others. We found that patients with events and matched controls had similar stroke and bleeding risk as estimated by CHA_2_DS_2_-VASc and HAS-BLED scores, respectively, despite not having been matched on these measures. This provides some evidence that the health status of the comparator groups was similar at the time of NVAF diagnosis. Given the long timeframe of the study and advanced age of NVAF patients, as well as their poor health status and the severity of the health outcomes being examined, we expected a substantial amount of loss-to-followup prior to the end of the 3-year timeframe of interest. Having at least 2 years of postevent followup for 44% and complete data for 29% of the study population is therefore, a strength of the study. However, the patients remaining after 3 years of followup may not be representative of the general NVAF cohort. We attempted to account for this by not requiring a minimum duration of followup after events and also including all patients with data available at each time point, not just patients with the complete data for all 3 years. Furthermore, because nondisabled patients eligible for Medicare are almost exclusively over the age of 65 years, data on younger NVAF patients were unavailable in this study.

In spite of stated limitations, the results of this study offer a unique insight into costs of stroke and hemorrhagic outcomes among NVAF for up to several years following the events. While the acute costs of events associated with NVAF and anticoagulation treatment were most dramatic in the first year, the total healthcare costs for patients with events that were alive and contributing data for up to 3 years remained elevated as compared to patients with NVAF who did not have these events. Thus, a proper cost-effectiveness assessment that takes into account the true long-term costs of stroke and major bleeding events is required when weighing the risks (bleeding) and benefits (stroke prevention) of anticoagulation in patients with NVAF. 

## Figures and Tables

**Table 1:** Study codes.

Condition	ICD-9 codes	CPT codes
Patient identification		
Atrial fibrillation	427.31	
Valvular conditions	Procedure codes: 35.0, 35.00–35.04, 35.1, 35.10–35.14, 35.2, 35.20–35.28, 35.96	33400, 33401, 33403, 33405, 33406, 33410–33415, 33417, 33420, 33422, 33425–33427, 33430, 33460, 33463–33465, 33468, 33470, 33472, 33474–33476, 33478, 33496, 33600, 33602
Outcomes		
Ischemic stroke	433.x1, 434.x1, 436, 437.1, 437.9	
Major bleeding		
Intracranial hemorrhage	430, 431, 432	
Other major bleeds	423.0, 455.2, 455.5, 455.8, 456.0, 456.2, 459.0, 530.7, 530.8, 531.0, 531.2, 531.4, 531.6, 532.0, 532.2, 532.4, 532.6, 533.0, 533.2, 533.4, 533.6, 534.0, 534.2, 534.4, 534.6, 562.0, 562.1, 569.3, 569.85, 578, 599.7, 626.2, 626.6, 719.1, 784.7, 786.3, 852, 853	
CHA_2_DS_2_-VASc components		
Cardiac failure	398.91, 402.x1, 404.x3, 425, 428	
Hypertension^1^	362.11, 401, 402, 403, 404, 405	
Diabetes mellitus^1^	250, 357.2, 362.0, 366.41	
Prior TIA^2^	362.34, 435	
Vascular disease	410, 411, 412, 413, 414, 440, 441, 442, 443, 444, 445 Procedure codes: 00.66, 36.0, 36.1, 39.25	33510–33545, 34051, 34151, 34201, 34203, 34800–34834, 34900, 35081–35103, 35131, 35132, 35141, 35142, 35151, 35152, 35331, 35341, 35351, 35355, 35361, 35363, 35371, 35372, 35381, 35450, 35452, 35454, 35456, 35459, 35470, 35471, 35472, 35473, 35474, 35480, 35481, 35482, 35483, 35485, 35490, 35491, 35492, 35493, 35495, 35521, 35531, 35533, 35541, 35546, 35546, 35548, 35549, 35551, 35556, 35558, 35563, 35565, 35566, 35571, 35583, 35585, 35587, 35621, 35623, 35646, 35647, 35651, 35654, 35656, 35661, 35663, 35665, 35666, 35671, 92980, 92981, 92982, 92984
HAS-BLED components		
Hypertension^1^	362.11, 401, 402, 403, 404, 405	
Abnormal renal function	582, 583, 585, 586	
Abnormal liver function	570, 571, 572, 573, 790.4	
Prior TIA^2^	362.34, 435	
Excessive alcohol use	291.0, 291.1, 291.2, 303, 305.0, 535.3, V11.3	

Note: With the exception of valvular conditions, ICD-9 codes reported as 3 digits will include all 4-digit and 5-digit codes beginning with the same 3 digits. For 4-digit codes, any 5-digit code beginning with the same 4 digits will also be included. ICD-9 codes reported are diagnosis codes unless otherwise indicated.

^1^Patient must have at least 2 diagnoses documented in the same calendar quarter to be considered as having the condition.

^2^Risk score definition includes prior stroke, but with the exclusion of patients with prior stroke, only prior TIA is applicable in this study.

**Table 2:** Matching characteristics of medicare patients with NVAF with and without stroke and bleeding events.

			Major bleeding events			
Matching characteristics		Ischemic stroke	ICH		Other major bleeds	
	Event *N* = 7,799	Control *N* = 33,084	Event *N* = 1,276	Control *N* = 5,097	Event *N* = 14,996	Control *N* = 60,058
Age at AF diagnosis, years						
Mean (SD)	81.1 (7.6)	81.1 (7.9)	79.9 (7.3)	79.9 (7.6)	80.0 (7.7)	79.8 (7.8)
Age, categorized, *n* (%)						
65–69 years	626 (8.0%)	2,676 (8.1%)	119 (9.3%)	482 (9.5%)	1,498 (10.0%)	6,282 (10.5%)
70–74 years	1,058 (13.6%)	4,569 (13.8%)	197 (15.4%)	815 (16.0%)	2,408 (16.1%)	9,916 (16.5%)
75–79 years	1,582 (20.3%)	6,647 (20.1%)	285 (22.3%)	1,156 (22.7%)	3,328 (22.2%)	13,537 (22.5%)
80–84 years	1,791 (23.0%)	7,318 (22.1%)	319 (25.0%)	1,235 (24.2%)	3,295 (22.0%)	12,696 (21.1%)
≥85 years	2,742 (35.2%)	11,874 (35.9%)	356 (27.9%)	1,409 (27.6%)	4,467 (29.8%)	17,621 (29.3%)
Gender, *n* (%)						
Male	2,913 (37.4%)	12,287 (37.1%)	634 (49.7%)	2,506 (49.2%)	6,507 (43.4%)	25,669 (42.7%)
Female	4,886 (62.7%)	20,797 (62.9%)	642 (50.3%)	2,591 (50.8%)	8,489 (56.6%)	34,389 (57.3%)
Race, *n* (%)						
White	7,194 (92.2%)	31,539 (95.3%)	1,191 (93.3%)	4,901 (96.2%)	14,018 (93.5%)	57,668 (96.0%)
Black	445 (5.7%)	1,012 (3.1%)	50 (3.9%)	120 (2.4%)	678 (4.5%)	1,628 (2.7%)
Hispanic	66 (0.9%)	202 (0.6%)	17 (1.3%)	29 (0.6%)	126 (0.8%)	306 (0.5%)
Other	85 (1.1%)	288 (0.9%)	16 (1.3%)	44 (0.9%)	156 (1.0%)	408 (0.7%)
Unknown	9 (0.1%)	43 (0.1%)	2 (0.2%)	3 (0.1%)	18 (0.1%)	48 (0.1%)
US geographic location, *n* (%)						
Northeast	1,547 (19.8%)	6,408 (19.4%)	251 (19.7%)	975 (19.1%)	3,251 (21.7%)	12,450 (20.7%)
South	1,970 (25.3%)	8,599 (26.0%)	317 (24.8%)	1,270 (24.9%)	4,151 (27.7%)	16,768 (27.9%)
Midwest	3,227 (41.4%)	14,034 (42.4%)	525 (41.1%)	2,160 (42.4%)	5,818 (38.8%)	24,257 (40.4%)
West	1,051 (13.5%)	4,043 (12.2%)	183 (14.3%)	692 (13.6%)	1,767 (11.8%)	6,570 (10.9%)
Other/unknown	4 (0.1%)	3 (0.0%)	0 (0.0%)	0 (0.0%)	9 (0.1%)	12 (0.0%)
Year of AF diagnosis, *n* (%)						
2000–2003	4,919 (63.1%)	20,082 (60.7%)	715 (56.0%)	2,651 (52.0%)	8,935 (59.6%)	33,254 (55.4%)
2004–2006	2,132 (27.3%)	9,449 (28.6%)	403 (31.6%)	1,700 (33.4%)	4,359 (29.1%)	18,786 (31.3%)
2007–2009	748 (9.6%)	3,553 (10.7%)	158 (12.4%)	746 (14.6%)	1,704 (11.4%)	8,018 (13.4%)
Duration of study enrollment, *n* (%)						
3–<6 months	1,571 (20.1%)	6,604 (20.0%)	342 (26.8%)	1,324 (26.0%)	2,123 (14.2%)	8,582 (14.3%)
6–<12 months	1,341 (17.2%)	5,671 (17.1%)	202 (15.8%)	839 (16.5%)	2,455 (16.4%)	9,838 (16.4%)
12–<18 months	933 (12.0%)	4,030 (12.2%)	138 (10.8%)	570 (11.2%)	1,812 (12.1%)	7,345 (12.2%)
18–<24 months	740 (9.5%)	3,160 (9.6%)	114 (8.9%)	468 (9.2%)	1,576 (10.5%)	6,354 (10.6%)
24–36 months	3,214 (41.2%)	13,621 (41.2%)	480 (37.6%)	1,896 (37.2%)	7,030 (46.9%)	27,939 (46.5%)
Warfarin use, *n* (%)						
Yes	2,568 (32.9%)	10,087 (30.5%)	532 (41.7%)	1,968 (38.6%)	5,594 (37.3%)	20,576 (34.3%)
No	5,231 (67.1%)	22,997 (69.5%)	744 (58.3%)	3,129 (61.4%)	9,402 (62.7%)	39,482 (65.7%)

ICH: intracranial hemorrhage; SD: standard deviation.

**Table 3:** Risk scores for Medicare patients with NVAF with and without stroke and bleeding events.

			Major bleeding events			
Matching characteristics		Ischemic stroke	ICH		Other major bleeds	
	Event *N* = 7,799	Control *N* = 33,084	Event *N* = 1,276	Control *N* = 5,097	Events *N* = 14,996	Control *N* = 60,058
CHA_2_DS_2_-VASc score						
Mean (SD)	3.89 (1.49)	3.81 (1.46)	3.72 (1.46)	3.68 (1.50)	3.91 (1.51)	3.69 (1.46)
Median (range)	4 (0–9)	4 (0–9)	4 (1–9)	4 (0–9)	4 (0–9)	4 (0–9)
CHA_2_DS_2_-VASc, categorized, *n* (%)						
0 points	7 (0.1%)	17 (0.1%)	0 (0.0%)	3 (0.1%)	13 (0.1%)	30 (0.1%)
1-2 points	1,353 (17.4%)	6,107 (18.5%)	272 (21.3%)	1,149 (22.5%)	2,647 (17.7%)	12,949 (21.6%)
3–5 points	5,337 (68.4%)	22,861 (69.1%)	858 (67.2%)	3,358 (65.9%)	10,086 (67.3%)	40,401 (67.3%)
6–9 points	1,102 (14.1%)	4,096 (12.4%)	146 (11.4%)	587 (11.5%)	2,249 (15.0%)	6,678 (11.1%)
HAS-BLED score						
Mean (SD)	1.33 (0.75)	1.30 (0.75)	1.32 (0.76)	1.27 (0.77)	1.33 (0.78)	1.25 (0.76)
Median (range)	1 (0–4)	1 (0–4)	1 (0–3)	1 (0–3)	1 (0–4)	1 (0–4)
HAS-BLED, categorized, *n* (%)						
0 points	1,036 (13.3%)	4,552 (13.8%)	181 (14.2%)	790 (15.5%)	2,099 (14.0%)	9,777 (16.3%)
1-2 points	6,481 (83.1%)	27,370 (82.7%)	1,042 (81.7%)	4,105 (80.5%)	12,139 (81.0%)	48,293 (80.4%)
3–5 points	282 (3.6%)	1,165 (3.5%)	53 (4.2%)	202 (4.0%)	757 (5.1%)	1,994 (3.3%)

ICH: intracranial hemorrhage; SD: standard deviation.

**Table 4:** Adjusted total incremental cost of ischemic stroke, intracranial hemorrhage, and other major bleeding events (2011 USD).

Matching characteristics	Ischemic stroke^1^ Adjusted cost (95% CI)	Major bleeding events	
		ICH^2^ Adjusted cost (95% CI)	Other major bleeds^2^ Adjusted cost (95% CI)
Acute and annual costs			
Acute (quarter of event)	$22,204 ($21,699–$22,808)	$33,887 ($31,692–$36,868)	$16,437 ($16,056–$16,853)
Year 1	$34,772 ($33,691–$35,870)	$49,216 ($45,490–$53,431)	$25,442 ($24,700–$26,190)
Year 2	$6,186 ($4,964–$7,450)	$8,572 ($5,207–$12,206)	$7,193 ($6,342–$8,038)
Year 3	$4,504 ($3,383–$5,617)	$3,150 ($475–$5,764)	$5,852 ($5,010–$6,671)

ICH: intracranial hemorrhage; CI: confidence interval.

Note: multivariate adjusted costs were estimated using generalized estimating equation (GEE) models with a gamma distribution and log link function. Year 1 costs include acute costs incurred during the quarter of the event.

^1^Adjusted for age group, gender, race, geographic region, year of NVAF diagnosis, warfarin use, cardiac failure, hypertension, diabetes, prior TIA, and vascular disease.

^2^Adjusted for age group, gender, race, geographic region, year of NVAF diagnosis, warfarin use, hypertension, abnormal renal function, abnormal liver function, and excessive alcohol use.

**Table 5:** Incremental utilization for the first, second, and third years following ischemic stroke, intracranial hemorrhage, and other major bleeding events (2011 USD).

Incremental utilization	Ischemic stroke			ICH			Other major bleeds		
	Year 1	Year 2	Year 3	Year 1	Year 2	Year 3	Year 1	Year 2	Year 3
Patients with events, *n *	4,887	3,214	2,121	732	480	310	10,417	7,030	4,682
Controls, *n *	20,811	13,620	8,968	2,934	1,896	1,218	41,634	27,936	18,563
Visits, mean (SD)									
Inpatient admissions	1.8 (1.9)	0.2 (2.1)	0.1 (1.5)	1.9 (2.0)	0.2 (2.1)	0.1 (1.3)	1.7 (2.0)	0.5 (2.0)	0.3 (1.8)
Inpatient length of stay	13.0 (18.8)	1.7 (17.3)	0.8 (12.1)	17.5 (21.1)	1.8 (17.0)	0.4 (9.4)	9.3 (16.7)	2.8 (14.9)	1.9 (12.6)
Outpatient visits	29.3 (50.9)	7.6 (54.5)	5.2 (45.6)	37.5 (55.6)	3.9 (52.1)	2.5 (44.6)	29.7 (53.3)	11.0 (57.6)	8.8 (48.5)
Costs, mean (SD)									
Total cost for patients with recurrence	$36,446 ($46,915)	$5,651 ($24,653)	$6,425 ($36,941)	$54,132 ($48,830)	$3,960 ($20,287)	$355 ($27,807)	$29,168 ($44,457)	$6,866 ($23,571)	$11,368 ($38,379)
Total cost for patients without recurrence	$32,434 ($43,046)	$2,391 ($21,493)	$3,349 ($31,199)	$47,091 ($54,879)	$3,957 ($23,026)	$3,434 ($28,414)	$22,608 ($43,323)	$2,908 ($22,163)	$4,253 ($33,263)
Inpatient	$16,669 ($25,592)	$1,793 ($27,022)	$665 ($16,337)	$26,904 ($38,184)	$2,314 ($27,144)	$294 ($12,219)	$12,723 ($27,637)	$2,791 ($27,077)	$2,248 ($18,982)
Outpatient	$3,869 ($11,865)	$689 ($12,584)	$426 ($10,366)	$6,952 ($13,924)	$1,520 ($14,808)	$765 ($13,663)	$4,703 ($13,612)	$2,081 ($14,468)	$1,635 ($12,115)
ER	$1,556 ($2,556)	$288 ($2,706)	$133 ($1,976)	$2,029 ($3,329)	$318 ($2,809)	$239 ($1,819)	$1,709 ($2,885)	$443 ($2,984)	$372 ($2,294)
SNF	$7,160 ($14,392)	$1,381 ($12,890)	$1,130 ($9,303)	$7,832 ($14,374)	$1,560 ($12,664)	$444 ($8,112)	$2,688 ($10,366)	$751 ($11,270)	$587 ($7,886)
Home healthcare	$3,043 ($11,375)	$918 ($9,640)	$666 ($8,275)	$2,676 ($8,553)	$697 ($9,373)	$408 ($5,518)	$1,226 ($9,188)	$531 ($10,779)	$244 ($6,961)
Hospice	$341 ($6,376)	$406 ($8,440)	$629 ($8,093)	$874 ($6,856)	$1,364 ($10,438)	$806 ($7,627)	$54 ($5,504)	$84 ($6,897)	$46 ($5,446)
DME	$262 ($2,406)	$147 ($2,491)	$126 ($1,976)	$374 ($2,661)	$138 ($2,746)	$199 ($2,588)	$309 ($3,225)	$256 ($3,412)	$268 ($3,983)

ICH: intracranial hemorrhage; SD: standard deviation; ER: emergency room; SNF: skilled nursing facility; DME: durable medical equipment.

Note: incremental utilization is calculated as the difference between the utilization measure for each patient with an event and matched controls during the given time interval. All patients with data at each time point are included in the estimates.

## References

[1] Naccarelli GV, Varker H, Lin J, Schulman KL (2009). Increasing prevalence of atrial fibrillation and flutter in the United States. *American Journal of Cardiology*.

[2] Kannel WB, Benjamin EJ (2008). Status of the epidemiology of atrial fibrillation. *Medical Clinics of North America*.

[3] Wolf PA, Abbott RD, Kannel WB (1991). Atrial fibrillation as an independent risk factor for stroke: the Framingham Study. *Stroke*.

[4] Singer DE, Albers GW, Dalen JE (2008). Antithrombotic therapy in atrial fibrillation: American College of Chest Physicians evidence-based clinical practice guidelines (8th edition). *Chest*.

[5] Darkow T, Vanderplas AM, Lew KH, Kim J, Hauch O (2005). Treatment patterns and real-world effectiveness of warfarin in nonvalvular atrial fibrillation within a managed care system. *Current Medical Research and Opinion*.

[6] Lakshminarayan K, Solid CA, Collins AJ, Anderson DC, Herzog CA (2006). Atrial fibrillation and stroke in the general medicare population: a 10-year perspective (1992 to 2002). *Stroke*.

[7] Boulanger L, Kim J, Friedman M, Hauch O, Foster T, Menzin J (2006). Patterns of use of antithrombotic therapy and quality of anticoagulation among patients with non-valvular atrial fibrillation in clinical practice. *International Journal of Clinical Practice*.

[8] Coyne KS, Paramore C, Grandy S, Mercader M, Reynolds M, Zimetbaum P (2006). Assessing the direct costs of treating nonvalvular atrial fibrillation in the United States. *Value in Health*.

[9] Boccuzzi SJ, Martin J, Stephenson J (2009). Retrospective study of total healthcare costs associated with chronic nonvalvular atrial fibrillation and the occurrence of a first transient ischemic attack, stroke or major bleed. *Current Medical Research and Opinion*.

[10] Engel-Nitz NM, Sander SD, Harley C, Rey GG, Shah H (2010). Costs and outcomes of noncardioembolic ischemic stroke in a managed care population. *Vascular health and risk management*.

[11] Wolowacz SE, Samuel M, Brennan VK, Jasso-Mosqueda JG, Van Gelder IC (2011). The cost of illness of atrial fibrillation: a systematic review of the recent literature. *Europace*.

[12] Miller PSJ, Andersson FL, Kalra L (2005). Are cost benefits of anticoagulation for stroke prevention in atrial fibrillation underestimated?. *Stroke*.

[13] Wang TJ, Massaro JM, Levy D (2003). A risk score for predicting stroke or death in individuals with new-onset atrial fibrillation in the community: the Framingham heart study. *Journal of the American Medical Association*.

[14] Birman-Deych E, Waterman AD, Yan Y, Nilasena DS, Radford MJ, Gage BF (2005). Accuracy of ICD-9-CM codes for identifying cardiovascular and stroke risk factors. *Medical Care*.

[15] Wahl PM, Rodgers K, Schneeweiss S (2010). Validation of claims-based diagnostic and procedure codes for cardiovascular and gastrointestinal serious adverse events in a commercially-insured population. *Pharmacoepidemiology and Drug Safety*.

[16] Gage BF, Birman-Deych E, Kerzner R, Radford MJ, Nilasena DS, Rich MW (2005). Incidence of intracranial hemorrhage in patients with atrial fibrillation who are prone to fall. *American Journal of Medicine*.

[17] White RH, Beyth RJ, Zhou H, Romano PS (1999). Major bleeding after hospitalization for deep-venous thrombosis. *American Journal of Medicine*.

[18] Ming K, Rosenbaum PR (2000). Substantial gains in bias reduction from matching with a variable number of controls. *Biometrics*.

[19] Rosenbaum PR (1989). Optimal matching for observational studies. *Journal of the American Statistical Association*.

[20] Miettinen OS (1969). Individual matching with multiple controls in the case of all-or-none responses. *Biometrics*.

[21] Ury HK (1975). Efficiency of case control studies with multiple controls per case: continuous or dichotomous data. *Biometrics*.

[22] Camm AJ, Kirchhof P, Lip GYH (2010). Guidelines for the management of atrial fibrillation: the task force for the management of atrial fibrillation of the european society of cardiology (esc). *European Heart Journal*.

[23] Beyth RJ, Quinn LM, Landefeld CS (1998). Prospective evaluation of an index for predicting the risk of major bleeding in outpatients treated with warfarin. *American Journal of Medicine*.

[24] Gage BF, Yan Y, Milligan PE (2006). Clinical classification schemes for predicting hemorrhage: results from the National Registry of Atrial Fibrillation (NRAF). *American Heart Journal*.

[25] Kuijer PMM, Hutten BA, Prins MH, Büller HR (1999). Prediction of the risk of bleeding during anticoagulant treatment for venous thromboembolism. *Archives of Internal Medicine*.

[26] Pisters R, Lane DA, Nieuwlaat R, De Vos CB, Crijns HJGM, Lip GYH (2010). A novel user-friendly score (HAS-BLED) to assess 1-year risk of major bleeding in patients with atrial fibrillation: the Euro heart survey. *Chest*.

[27] Shireman TI, Mahnken JD, Howard PA, Kresowik TF, Hou Q, Ellerbeck EF (2006). Development of a contemporary bleeding risk model for elderly warfarin recipients. *Chest*.

[28] Lip GYH, Frison L, Halperin JL, Lane DA (2011). Comparative validation of a novel risk score for predicting bleeding risk in anticoagulated patients with atrial fibrillation: the HAS-BLED (hypertension, abnormal renal/liver function, stroke, bleeding history or predisposition, labile INR, elderly, drugs/alcohol concomitantly) score. *Journal of the American College of Cardiology*.

[29] Diehr P, Yanez D, Ash A, Hornbrook M, Lin DY (1999). Methods for analyzing health care utilization and costs. *Annual Review of Public Health*.

[30] Mercaldi CJ, Ciarametaro M, Hahn B (2011). Cost efficiency of anticoagulation with warfarin to prevent stroke in medicare beneficiaries with nonvalvular atrial fibrillation. *Stroke*.

[31] Caro JJ, Huybrechts KF (1999). Stroke treatment economic model (STEM): predicting long-term costs from functional status. *Stroke*.

[32] Dewey HM, Thrift AG, Mihalopoulos C (2003). Lifetime cost of stroke subtypes in Australia: findings from the North East Melbourne Stroke Incidence Study (NEMESIS). *Stroke*.

[33] Youman P, Wilson K, Harraf F, Kalra L (2003). The economic burden of stroke in the United Kingdom. *PharmacoEconomics*.

